# Valuation in major depression is intact and stable in a non-learning environment

**DOI:** 10.1038/srep44374

**Published:** 2017-03-10

**Authors:** Dongil Chung, Kelly Kadlec, Jason A. Aimone, Katherine McCurry, Brooks King-Casas, Pearl H. Chiu

**Affiliations:** 1 Virginia Tech Carilion Research Institute, Roanoke, VA, USA; 2 Department of Electrical and Computer Engineering, Virginia Tech, Blacksburg, VA, USA; 3 School of Neuroscience, Virginia Tech, Blacksburg, VA, USA; 4 Department of Economics, Baylor University, Waco, TX, USA; 5 Department of Psychology, Virginia Tech, Blacksburg, VA, USA.

## Abstract

The clinical diagnosis and symptoms of major depressive disorder (MDD) have been closely associated with impairments in reward processing. In particular, various studies have shown blunted neural and behavioral responses to the experience of reward in depression. However, little is known about whether depression affects individuals’ valuation of potential rewards during decision-making, independent from reward experience. To address this question, we used a gambling task and a model-based analytic approach to measure two types of individual sensitivity to reward values in participants with MDD: ‘risk preference,’ indicating how objective values are subjectively perceived, and ‘inverse temperature,’ determining the degree to which subjective value differences between options influence participants’ choices. On both of these measures of value sensitivity, participants with MDD were comparable to non-psychiatric controls. In addition, both risk preference and inverse temperature were stable over four laboratory visits and comparable between the groups at each visit. Neither valuation measure varied with severity of clinical symptoms in MDD. These data suggest intact and stable value processing in MDD during risky decision-making.

Major depressive disorder (MDD) has been associated with impairments in reward processing, and many studies indicate that symptoms of MDD correlate with diminished neural and behavioral responses when rewards are presented^1^,^2^,^3^,^4^,^5^,^6^. These studies have typically used reward learning and other tasks that provide feedback about rewards and focused on individuals’ responses at this feedback or ‘reward outcome’ phase (see Rizvi *et al*.^7^ for review). However, little is known about how depression affects reward valuation during decision-making in the absence of learning and feedback. Understanding whether individuals with MDD have disrupted valuation during decision-making at the ‘decision phase’, separate from reward outcome, will clarify whether individuals with MDD are disrupted overall in reward valuation or more specifically in experiencing rewards. Here, we used a risky decision-making task, a model-based analytic approach, and a repeated measures within-subject design across four visits to investigate whether participants with MDD have intact or disrupted valuation during decision-making in the absence of learning and feedback.

Sixty-nine individuals with current MDD and 41 non-psychiatric controls were recruited for the current study. To investigate ‘value sensitivity’ during decision-making independent from feedback, we asked participants to complete a risky decision-making task (adapted from Holt & Laury^8^ and Dickhaut *et al*.^9^) (Fig. 1). During the task, participants made a series of nine choices between two gambles, one of which was objectively riskier than the other^8^. Each pair of gambles had the same high- and low-payoff probabilities; the high-payoff probability increased from 10% to 90% in 10% increments (as the low-payoff probability decreased) along the nine pairs. Participants’ choices between the safer and riskier options, at each payoff and probability combination, were recorded to investigate individual value sensitivity. Participants were paid based on the actual outcome of one of their choices; the outcome was determined after all choices had been made (i.e., no feedback at each decision). This paradigm allowed us to examine valuation during decision-making, independent from potential learning and outcome effects.

Tasks of this sort are classically used to study individuals’ value-based decision-making under risk^10^,^11^, and expected utility theory^12^ points to two basic components that account for differences among individuals’ choices in such tasks. The first, ‘risk preference^13^,^14^ (RP)’ reflects how objective values are subjectively perceived (subjective value) and is quantified by the curvature of a power utility function^12^. This approach is equivalent to defining risk as the variance of each option and thus, takes into account both the reward magnitude outcome spread and probability information. The second component determines the degree to which subjective value differences between options affect the probability of choosing one option over the other, and is often referred to as ‘inverse temperature^15^ (IT)’. Both components characterize individual differences in the direction and the degree to which objective values impact individual choices, and thus are used as measures of *value sensitivity* in the current study. Note that each measure explains a different functional relationship between subjective values and decision-making: RP accounts for nonlinear (concave or convex function) subjective valuation and IT is a linear scaling of subjective values (similar to ‘reward sensitivity’ in other MDD studies^1^; see Methods for expected utility model specifications). The value sensitivity measures were estimated from individuals’ choices using maximum a posteriori fitting (see Methods for parameter estimation procedure).

Participants completed the decision-making task on up to four laboratory visits as part of a longitudinal study; on average, visits were separated by 5.5 weeks (mean of 116.27 days between Time 1 and Time 4 visits). At each visit, participants were instructed that one of their actual choices would be randomly selected and played out to determine their payoff at the end of the visit. The payoff was determined by the values of a gamble selected via random number generator from the participant’s actual choices and a roll of a hundred-sided die (the first determining which gamble would be played and the second determining the payoff). Participants who made less than two visits to the laboratory, had Beck Depression Inventory (BDI-II) scores^16^ >12 for controls or <13 at Time 1 for MDD participants, or always chose the option with smaller expected value were excluded from analyses (see Methods for numbers of excluded participants for each criterion). The analyzed sample for RP and IT parameter estimation included 33 non-psychiatric controls (14 females; age = 33.00 ± 11.31) and 65 individuals with MDD (48 females; age = 37.92 ± 11.48). See Table 1 for further demographic information.

## Results

### Valuation is comparable between MDD and non-psychiatric control participants

To compare value sensitivity in MDD participants with that of non-psychiatric controls, we estimated each individual’s risk preference and inverse temperature for each visit, and first compared the means of these parameters between groups (see Methods for details about parameter estimation). Thus, RP and IT at each of the four visits were computed for each individual, for participants who visited all four times (*N*_control_ = 28, *N*_MDD_ = 47). Group mean parameter values were: RP_control_ = 0.50 ± 0.31; RP_MDD_ = 0.46 ± 0.31; IT_control_ = 3.41 ± 0.41; and IT_MDD_ = 3.25 ± 0.43 (mean ± s.d.). Note that both the MDD and non-psychiatric control groups showed risk aversion (RP < 1) consistent with Holt & Laury^8^. Across four laboratory visits, participants with MDD showed comparable RP and IT to that of non-psychiatric controls (Fig. 2ai,bi; RP: F(1, 219) = 0.63, *P* = 0.43; IT: F(1, 219) = 2.68, *P* = 0.11; Group × Time mixed-design ANOVAs with rank transformation^17^). These results were confirmed using Bayesian null hypothesis significance testing (JASP, version 0.8.0.0^18^) which similarly indicated no group differences in RP or IT (see Supplementary Text for additional details and Table S5 for Bayes factors). These results indicate that MDD and non-psychiatric control participants have comparable linear and nonlinear value sensitivities during decision-making.

### Valuation is stable over time for both MDD and non-psychiatric control participants

Previous studies have shown that risk preferences measured with variations of the Holt & Laury task^8^ are stable over time in unselected control individuals, particularly when model-based estimates were used^19^,^20^. As an initial test of RP and IT stability within MDD and control participants, we compared within-group means over time; these analyses indicate that neither parameter differed over time for either group (RP_control_: **χ**^**2**^(3, 81) = 2.12, *P* = 0.55; RP_MDD_: **χ**^**2**^(3, 138) = 0.66, *P* = 0.88; IT_control_: **χ**^**2**^(3, 81) = 2.94, *P* = 0.40; IT_MDD_: **χ**^**2**^(3, 138) = 2.40, *P* = 0.49; Friedman’s tests, analogous to non-parametric repeated measures ANOVAs; only participants who visited all four times were examined here (*N*_control_ = 28, *N*_MDD_ = 47)). Adopting the approach of previous studies for measuring temporal stability, we also examined the stability of RP and IT within controls and participants with MDD by correlating the value of each parameter between pairs of visits ([1^st^ vs 2^nd^ visit], [1^st^ vs 3^rd^ visit], … [3^rd^ vs 4^th^ visit]) (Fig. 2aii,bii). Both control and MDD participants showed moderate to high stability in both RP and IT, respectively (mean correlation coefficients: RP_control_: Spearman ρ = 0.57; RP_MDD_: ρ = 0.54; IT_control_: ρ = 0.48; IT_MDD_: ρ = 0.57; see Fig. 2aii and bii for full correlation matrix; Supplementary Table S2 for sample size at each visit). Note that the proportion of risky choices, a model-free measure of risk preference, was also stable over time in both the MDD and control groups (see Supplementary Fig. S2 for model-free risk preference stability over time). Again, Bayesian null hypothesis significance testing confirmed the stable RP and IT across visits (see Supplementary Text and Table S5 for details and Bayes factors). These significant correlations indicate that for MDD and control participants, the risk preference and inverse temperature measures of value sensitivity at the decision phase are stable over time.

### Valuation does not vary with severity of clinical symptoms in MDD

Given previous reports that reward sensitivity at decision outcome varies with symptoms in depression^1^,^21^,^22^,^23^, we also examined whether RP and IT varied systematically with depressive or anxious symptoms. Symptoms were measured using the BDI-II^16^, Anhedonia subscale of the BDI-II (sum of BDI items 4, 12, 15, and 21)^24^, State Anxiety Scale of the Spielberger State-Trait Anxiety Inventory (STAI)^25^, and the five subscales of the Mood and Anxiety Symptoms Questionnaire (MASQ; Anhedonic Depression, Anxious Arousal, General distress (GD):Anxiety, GD:Depression, and GD:Mixed)^26^; correlations were performed within the MDD group. None of the clinical symptom scores or changes in symptoms over time were related to MDD participants’ RP or IT parameter values (see Supplementary Fig. S1, Supplementary Table S3, and S4 for statistical test results). These data demonstrate that individual differences in value sensitivity during decision-making are not explained by clinical characteristics of MDD.

## Discussion

The current study used a risky decision-making task to investigate MDD individuals’ value sensitivity at the decision phase independent from learning and feedback. The within-subjects repeated-measures design allowed us to examine the stability of the value sensitivity measures, and the model-based approach dissociated linear (inverse temperature) and nonlinear (risk preference) value sensitivities that together determine behavioral choices during risky decision-making.

A few previous studies have used risky decision-making paradigms and measured MDD individuals’ risk preferences. The results, however, have been inconsistent. Some studies have reported decreased risk seeking behavior in individuals with MDD^21^,^27^,^28^, while other studies have reported comparable risk preferences between individuals with MDD and healthy individuals^29^,^30^. In the current study, we showed that risk preferences (nonlinear value sensitivity) in individuals with MDD are comparable with those of healthy individuals. The stability of risk preferences was tested across four repeated visits, and consistent with previous findings in unselected control individuals^19^,^20^,^31^, MDD participants showed stable risk preferences over time (c.f., model-free measures showing lower reliability^32^,^33^,^34^). In addition to estimating risk preference, we examined inverse temperature (linear value sensitivity, similar to ‘reward sensitivity’ in other MDD studies^1^) at the decision phase, and showed that MDD participants have stable and comparable inverse temperature compared with non-psychiatric controls. In addition, none of the clinical symptom severity measures within participants with MDD were related to individual differences in risk preference or inverse temperature. These results indicate that in contrast with previous decision-making studies showing blunted valuation at the outcome phase in MDD^1^, neither linear nor nonlinear value sensitivity at the decision phase in MDD was different from that of controls.

To date, studies examining valuation in MDD have focused primarily on the outcome phase of reward learning tasks and shown impaired valuation, including diminished neural reward responses^35^,^36^,^37^, reduced learning rate^38^, lower reward sensitivity^1^, or enhanced exploration (more frequent choice shifting)^39^,^40^ in participants with MDD. A few other studies have used various non-learning tasks and have suggested that individuals with MDD have low motivation for monetary reward^21^,^41^,^42^; however, in these studies, the focus was also on responses at the outcome phase^22^,^23^. Unlike the abundant literature about responses to reward outcome (particularly during reward learning), little is known about whether individuals with MDD have intact ability to process and compare values during decision-making when no learning is required. The current study provided no outcome feedback during the task and thus focused on the decision phase dissociated from learning and reward experience. These data showed that during the decision phase, participants with MDD have value processes comparable to that of healthy individuals. This is consistent with previous studies showing intact neural responses in individuals with MDD during reward anticipation (prior to outcome)^43^,^44^. Together, the present data suggest that individuals with MDD have intact valuation when reward contingencies are fully known (no reward learning required) and suggest that previously reported valuation deficits in MDD are specific to the outcome phase of tasks in which rewards are experienced and learning occurs. We note that this conclusion is drawn based on the assumption of linear probability weighting (see Supplementary Text for nonlinear probability weighting function) and the present task where each pair of gambles had the same high- and low-payoff probabilities. Individuals with MDD may exhibit altered valuation in other environments (e.g., when making choices between two options that have different payoff probabilities); these possibilities cannot be ruled out in the present data.

In MDD, intact valuation, dissociated from learning, may provide mechanistic insight about behavioral activation therapies for depression^45^. These type of therapies engage individuals with potential positive reinforcers (rewards) in a structured manner and, in doing so, allow individuals with MDD to largely bypass disrupted learning processes. That is, behavioral activation provides a guided learning environment wherein engagement and experience of action-reward contingencies are enforced, allowing for the value of rewards to evolve from being unsampled and ambiguous to sampled and fully known. Once these values are known, intact decision processes such as those identified here allow individuals to engage in healthy choices. As our data indicate, when action-reward contingencies are fully known, participants with MDD show intact valuation during decision-making. We speculate that this state is comparable to the endpoint of successful behavioral activation wherein the experience of reward is restored. In brief summary, the current study suggests specificity of previously reported value processing disruptions in MDD, informs the conditions under which sensitivity to reward values is preserved, and offers the possibility that learning about reward values, rather than discriminating among values when making decisions, may be a mechanistic target for intervention in MDD.

## Methods

### Participants

Fifty non-psychiatric controls and 80 individuals with MDD were recruited as part of a larger ongoing study examining neural substrates of treatment response in MDD (neural and treatment data will be analyzed as part of another manuscript). Among these participants, we included individuals who at least participated in both Time 1 and 4 laboratory visits to maximize the time interval for test-retest reliability. These inclusion criteria yielded 41 non-psychiatric controls and 69 individuals with MDD for the present study. Basic inclusion/exclusion criteria were initially assessed via telephone and were confirmed during the first laboratory visit using the Structured Clinical Interview for DSM-IV-TR Axis I Disorders – Research Version – Patient Edition (With Psychotic Screen) (SCID-I/P)^46^ and selected modules of the Mini-International Neuropsychiatric Interview (M.I.N.I.)^47^. At study intake, individuals in the MDD group met DSM-IV criteria for MDD and/or dysthymia while individuals in the control group did not meet criteria for any current Axis I disorder. Exclusion criteria for all participants included contraindications to magnetic resonance imaging (MRI) and history of neurological disease. Following the initial screening visit (Time 1), participants returned to the lab up to three times; on average, there were 5.5 weeks between each visit. Three controls whose BDI-II scores were above the non-depressive range (i.e., greater than 12) at any visit and two individuals with MDD who had BDI-II scores in the non-depressive range (i.e., less than 13) at Time 1 were additionally excluded from analyses^48^. Five controls and two individuals with MDD who always chose the option with smaller expected value were also excluded. Therefore, the analyzed sample for RP and IT parameter estimation included 33 healthy controls (14 females; age = 33.00 ± 11.31) and 65 participants with MDD (48 females; age = 37.92 ± 11.48). See Table 1 for additional demographic information. Use of psychotropic medication was not an exclusion criterion for individuals with MDD, and at study enrollment, 20 participants with MDD were taking one or more psychotropic medications. As noted above, these data were collected as part of a larger study examining neural substrates of treatment response in MDD, and a subgroup of individuals with MDD received cognitive behavioral therapy (CBT) over the course of participation (N = 45; see Supplementary Table S1 for demographic data for the treatment group). As such, participants showed decline in symptoms over time; neither symptoms nor symptom change was related to either RP or IT (as detailed throughout the main text). All participants provided written informed consent following an explanation of study procedures. The study was approved by the Institutional Review Board (IRB) of Virginia Tech, and all experimental procedures followed relevant institutional guidelines and regulations.

### Experimental procedure

**Figure 1:**
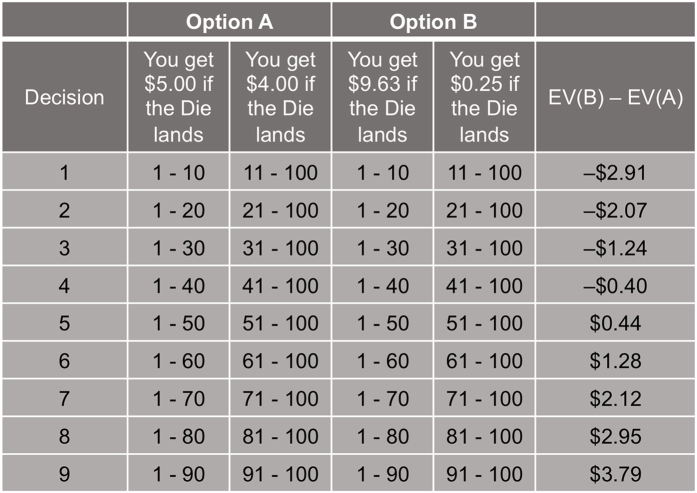
Payoffs and probabilities of paired gambles. Participants played a gambling task that consisted of a menu of probabilities of high- and low-payoff values. As per Holt & Laury^8^, participants made nine choices between two risky gambles ‘Option A’ and ‘Option B’. The high- and low-payoff assigned to each option were fixed as shown here. The probability associated with payoff values was represented as a range of numbers; this allowed participants to easily match the probability of each outcome with a roll of a hundred-sided die; this roll was performed after the task for one randomly selected gamble to determine the final outcome for payoff. The rightmost column shows the expected value differences between the Option A and B. Expected utility theory predicts that a risk neutral individual will choose Option A in decisions 1–4 where EV(B) <EV(A) and Option B in decisions 5–9 where EV(B) > EV(A).

**Figure 2:**
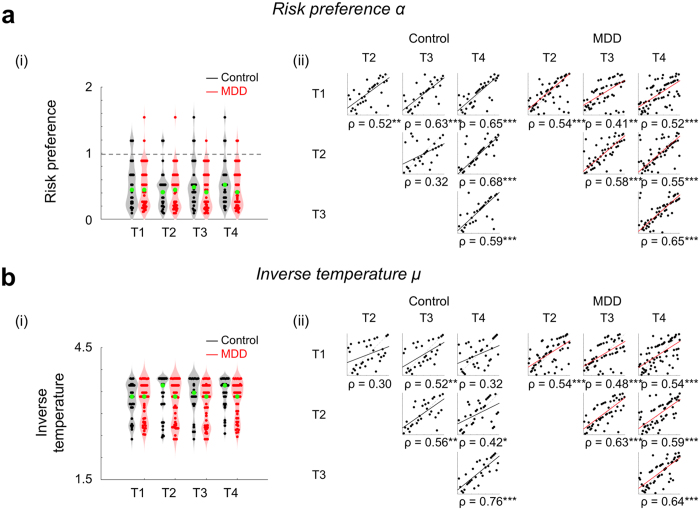
Estimated value sensitivities are comparable between non-psychiatric controls and individuals with MDD, and stable across visits. We used a standard power utility function and softmax choice rule to identify separate ‘risk preference’ and ‘inverse temperature’ parameters to explain nonlinear and linear value sensitivities in decision-making. (**ai, bi**) Estimated RP and IT were stable across four repeated visits for both MDD and control participants. Across the repeated visits, both RP and IT were comparable between the control and MDD groups (no main effect of group using mixed-design ANOVA with rank transformation). The gray dotted line indicates risk neutrality (RP = 1). Each point represents an individual participant; group medians are indicated in green. Gray and red shades show the distribution of data points along the y-axis. (**aii**, **bii**) Spearman’s correlation coefficients were calculated to test whether the rank order of the parameters among individuals was consistent between visits to the lab (([1^st^ vs 2^nd^ visit], [1^st^ vs 3^rd^ visit], ... [3^rd^ vs 4^th^ visit]). See Supplementary Table S2 for statistical results. Each point represents an individual participant, and the color-coded lines are the robust regression line between measures from two visits. The x- and y-axes each represent the rank order of individual participants at each visit (for simplicity, not labeled here); *P < 0.05, **P < 0.01, ***P < 0.001, uncorrected; all correlations were significant after applying multiple comparison correction (FDR *q* < 0.0001).

Participants made a series of nine choices between two gambles, one of which was objectively riskier than the other (adapted from Holt & Laury^8^) (Fig. 1). Each pair of gambles had the same high- and low-payoff probabilities that varied from 10% to 90% in 10% increments along the nine pairs. Payoff spreads between high- and low- payoffs were fixed for each option; ‘Option A’ had $5.00 and $4.00, and ‘Option B’ had $9.63 and $0.25 as potential payoffs. Participants were paid based on the actual outcome of one of their choices; the payoff was determined by the values of a gamble selected via random number generator from the participant’s actual choices and a roll of a hundred-sided die (the first determining which gamble would be played and the second determining the payoff).

### Model-free analyses

For model-free behavioral analyses, the proportion of choices of the risky option (P(risky)) among the nine pairs of gambles was used as a measure of risk preference. Given the expected value (EV) between pairs of choices (Fig. 1), a risk neutral individual should show P(risky) = 5/9 ≈ 0.56 (as per expected utility theory, a risk neutral individual is expected to choose Option B in the trials where EV(B) > EV(A), decisions 5–9, and to choose Option A in the trials where EV(B) < EV(A)). Higher P(risky) thus indicates risk seeking. P(risky) was calculated per visit and used to examine stability of model-free risk preferences over time in each group.

### Estimates of individual risk preference

We applied expected utility theory^12^ to estimate each individual’s risk preference (RP) and inverse temperature (IT) that predict the individual’s choices. We used a standard power utility function and softmax choice rule as described below:


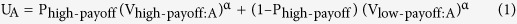



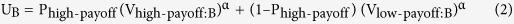



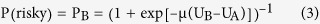


where U_A_ (U_B_) is the utility of the Option A (Option B), P is the probability of earning a payoff, V represents the payoff amount for each gamble, α is the *risk preference*, and μ is the *inverse temperature*. The estimated RP, α, indicates whether an individual is risk averse (0 < α < 1), risk neutral (α = 1), or risk seeking (α > 1). The estimated IT, μ, indicates how sensitive an individual is to the utility differences between the two gambles; larger μ indicates higher sensitivity to utility differences and μ ≈ 0 indicates utility (subjective value) insensitivity.

To achieve a more stable parameter estimation for each individual, we adopted a hierarchical model structure of the population^49^ in which it is assumed that a participant *i*’s parameters (μ_*i*_ and α_*i*_) are sampled from the population’s parameter distribution. Of importance, both controls and participants with MDD were considered to share the same group-level (population) distribution (equal prior), which allowed us to compare the two participant groups in the further analyses. That is, the two groups were assumed to be coming from the same distribution every time the group-level parameters were updated throughout the estimation procedures. As this approach may bias against finding group differences (because individuals from two groups are treated as samples from equal priors), we also implemented two additional approaches with different assumptions: i) separately estimating group-level distributions (biasing toward finding group differences) and ii) estimating an additional variable that captures a potential group mean difference (examining whether the mean group difference is different from zero) (see Supplementary Text for details).

Based on the assumptions for each approach, we estimated the group-level parameter distribution for each parameter and set the distribution as a prior for individual estimation (maximum a posteriori (MAP) estimation). In the current study, we set the group-level distribution of each parameter as a gamma distribution^50^ with a shape parameter, k, and a scale parameter, θ, (μ ~ Γ(k_μ_, θ_μ_); and α ~ Γ(k_α_, θ_α_)). For each iteration of the group-parameter estimation (max iteration of 15,000), 100 random samples were drawn from each parameter distribution for each participant, and the average of the calculated likelihoods was used as an approximation of the integral in the following equation:


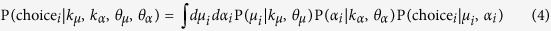



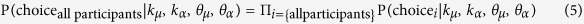


Note that all participants visited at least twice, including the 1^st^ and the 4^th^ visits.

To allow for RP and IT values to vary across time independent from subject-level information, we did not provide any information about subjects’ identity in the estimation step; that is, behavioral choices from a participant’s 1^st^ and 4^th^ visits were considered decision patterns from two independent participants. Note that estimated value sensitivities for the same subject from repeated visits were considered as repeated-measures for post estimation stability testing. To apply this method, we used 196 sets of behavioral choices for the group-level parameter estimation ([33 HC + 65 MDD] × [1^st^ visit + 4^th^ visit]; only 1^st^ and 4^th^ visits were used to provide an equal amount of choice information from each individual participant). The group-level parameters were used to define each parameter’s prior distribution for individual-level estimation, which was equally applied to individual-level estimations for all four visits. We fit the data using MAP, with the posterior function below:


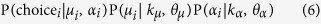


All parameter estimations were conducted with custom MATLAB R2015b (MathWorks) scripts and the fminsearch function in MATLAB with multiple initial values.

### Clinical measures

At each visit, participants completed a battery of self-report measures to assess current depression and anxiety symptoms. Depressive symptom severity was measured using the BDI-II, Anhedonia subscale of the BDI-II (sum of BDI items 4, 12, 15, and 21)^24^, and the Anhedonic Depression subscale score of the MASQ. Anxiety symptom severity was measured using the State Anxiety Scale of the STAI (State-Trait Anxiety Inventory) and the Anxious Arousal subscale of the MASQ. Additionally, general distress (GD) related to depressive symptoms, anxious symptoms, or a mixture of the two were measured using the MASQ subscales, GD: Anxiety, GD: Depression, and GD: Mixed, respectively.

### Statistical analyses

We examined if model-free risk preference (proportion risky choices) and model-based measures of value sensitivity (inverse temperature and risk preference) were consistent across multiple visits. IT and RP measures in both participant groups were not normally distributed (Shapiro-Wilk test *P* < 0.01 for IT and RP in each group and in each visit), and thus non-parametric tests were used as appropriate and available. First, to compare the means of IT and RP across four laboratory visits and between groups, we used mixed-design ANOVAs where visit number (Time 1, Time 2, Time 3, Time 4) was the within-subject factor and diagnostic group (MDD, control) was the between-subject factor. Parameters were first rank transformed and then inserted for the mixed-design ANOVAs^17^. In addition, we used Friedman’s test to examine whether IT and RP across four visits were stable or not, within each group. Second, Spearman’s correlations between risk preference measures from two different visits (‘1^st^ visit’ (T1) vs T2, T1 vs T3, T1 vs T4, T2 vs T3, T2 vs T4, and T3 vs T4) were calculated to test if the rank-order of risk preference within each group was consistent across multiple visits. All statistical tests were two-sided. False discovery rate (FDR) adjusted *q*-values where indicated were reported for multiple comparisons^51^. MATLAB R2015b was used for all statistical tests.

## Additional Information

**How to cite this article:** Chung, D. *et al*. Valuation in major depression is intact and stable in a non-learning environment. *Sci. Rep.* **7**, 44374; doi: 10.1038/srep44374 (2017).

**Publisher's note:** Springer Nature remains neutral with regard to jurisdictional claims in published maps and institutional affiliations.

## Supplementary Material

Supplementary Information

## Figures and Tables

**Table 1:** Demographic and symptom data.

	Control (*N* = 33)	Major depression (*N* = 65)
Male/female participants	14/19	17/48
Age (years)	33.00 ± 11.31	37.92 ± 11.48
Verbal intelligence quotient^a^	105.15 ± 14.90	107.06 ± 12.18
BDI-II		
Time 1	1.55 ± 2.05 (33)	30.71 ± 7.75 (65)
Time 2	1.40 ± 2.19 (30)	22.83 ± 10.70 (53)
Time 3	1.28 ± 1.79 (29)	19.12 ± 12.79 (49)
Time 4	1.64 ± 2.57 (33)	17.14 ± 13.34 (64)
BDI-II Anhedonia subscale^b^		
Time 1	0.39 ± 0.83 (33)	6.32 ± 2.23 (65)
Time 2	0.42 ± 1.03 (31)	4.74 ± 2.72 (53)
Time 3	0.24 ± 0.64 (29)	3.79 ± 2.97 (52)
Time 4	0.39 ± 0.86 (33)	3.45 ± 2.84 (65)
State Anxiety Scale		
Time 1	28.48 ± 6.55 (33)	49.05 ± 10.82 (65)
Time 2	27.39 ± 6.35 (31)	46.47 ± 10.97 (53)
Time 3	27.86 ± 7.72 (29)	42.04 ± 12.40 (51)
Time 4	27.79 ± 6.73 (33)	39.52 ± 12.76 (64)
MASQ subscales		
Anhedonic Depression		
Time 1	45.09 ± 8.97 (33)	83.11 ± 9.12 (65)
Time 2	43.29 ± 9.84 (31)	71.63 ± 14.39 (52)
Time 3	42.86 ± 10.72 (29)	66.47 ± 18.96 (51)
Time 4	43.38 ± 10.36 (32)	65.00 ± 16.86 (65)
Anxious Arousal		
Time 1	18.55 ± 1.99 (33)	26.85 ± 7.25 (65)
Time 2	18.39 ± 1.87 (31)	23.79 ± 6.90 (52)
Time 3	18.69 ± 3.29 (29)	23.80 ± 8.31 (51)
Time 4	18.28 ± 1.49 (32)	23.62 ± 8.21 (65)
GD: Anxiety		
Time 1	14.48 ± 2.59 (33)	25.03 ± 6.97 (65)
Time 2	13.42 ± 2.47 (31)	20.81 ± 6.18 (52)
Time 3	14.00 ± 2.60 (29)	20.59 ± 7.57 (51)
Time 4	13.44 ± 2.06 (32)	18.95 ± 6.86 (65)
GD: Depression		
Time 1	15.72 ± 2.82 (33)	38.94 ± 8.91 (65)
Time 2	15.16 ± 2.27 (31)	31.10 ± 9.57 (52)
Time 3	15.07 ± 3.62 (29)	27.49 ± 11.08 (51)
Time 4	15.09 ± 2.44 (32)	26.68 ± 11.74 (65)
GD: Mixed		
Time 1	22.55 ± 4.49 (33)	45.91 ± 8.41 (65)
Time 2	21.03 ± 4.53 (31)	39.08 ± 9.13 (52)
Time 3	20.79 ± 3.80 (29)	37.00 ± 11.01 (51)
Time 4	21.38 ± 4.83 (32)	34.55 ± 11.16 (65)

BDI-II, Beck Depression Inventory, Second Edition; State Anxiety Scale, State Anxiety Scale of the State-Trait Anxiety Inventory; MASQ, Mood and Anxiety Symptom Questionnaire; GD, General Distress; ^a^Verbal intelligence quotient scores were measured with the Wechsler Test of Adult Reading (WTAR); ^b^The Anhedonia subscale was created by summing responses on the following BDI-II items associated with anhedonia symptoms: loss of pleasure (item 4), loss of interest (item 12), loss of energy (item 15), and loss of interest in sex (item 21). Numbers of participants who were included for calculating mean and standard deviation of each questionnaire score are noted in parentheses; see Supplementary Fig. S1 for the BDI-II, State Anxiety Scale, and MASQ scores in the smallest subset of included participants (visiting all four times), and see Methods for details about inclusion criteria.

## References

[b1] Huys Q. J., Pizzagalli D. A., Bogdan R. & Dayan P. Mapping anhedonia onto reinforcement learning: a behavioural meta-analysis. Biology of mood & anxiety disorders 3, 1 (2013). 23782813 10.1186/2045-5380-3-12 PMC3701611

[b2] American Psychiatric Association Diagnostic and statistical manual of mental disorders: DSM-IV-TR. (American Psychiatric Press, Washington, DC., 2000).

[b3] Nelson B. D., Perlman G., Klein D. N., Kotov R. & Hajcak G. Blunted Neural Response to Rewards as a Prospective Predictor of the Development of Depression in Adolescent Girls. American Journal of Psychiatry, appi. ajp. 2016.15121524 (2016). 10.1176/appi.ajp.2016.15121524 27363510

[b4] Pizzagalli D. A., Iosifescu D., Hallett L. A., Ratner K. G. & Fava M. Reduced hedonic capacity in major depressive disorder: evidence from a probabilistic reward task. Journal of psychiatric research 43, 76–87 (2008). 18433774 10.1016/j.jpsychires.2008.03.001 PMC2637997

[b5] Weinberg A., Liu H., Hajcak G. & Shankman S. A. Blunted neural response to rewards as a vulnerability factor for depression: Results from a family study (2015). 10.1037/abn0000081 PMC4658279 26214708

[b6] Treadway M. T. & Zald D. H. Reconsidering anhedonia in depression: lessons from translational neuroscience. Neuroscience & Biobehavioral Reviews 35, 537–555 (2011). 20603146 10.1016/j.neubiorev.2010.06.006 PMC3005986

[b7] Rizvi S. J., Pizzagalli D. A., Sproule B. A. & Kennedy S. H. Assessing anhedonia in depression: Potentials and pitfalls. Neuroscience & Biobehavioral Reviews 65, 21–35 (2016). 26959336 10.1016/j.neubiorev.2016.03.004 PMC4856554

[b8] Holt C. A. & Laury S. K. Risk aversion and incentive effects. American Economic Review 92, 1644–1655 (2002).

[b9] Dickhaut J., Houser D., Aimone J. A., Tila D. & Johnson C. High stakes behavior with low payoffs: Inducing preferences with Holt–Laury gambles. Journal of Economic Behavior & Organization 94, 183–189 (2013).

[b10] Levy I., Snell J., Nelson A. J., Rustichini A. & Glimcher P. W. Neural representation of subjective value under risk and ambiguity. Journal of neurophysiology 103, 1036–1047 (2010). 20032238 10.1152/jn.00853.2009

[b11] O’Dhaniel A., Leong R. L. & Kurnianingsih Y. A. Cognitive fatigue destabilizes economic decision making preferences and strategies. PloS one 10, e0132022 (2015). 26230404 10.1371/journal.pone.0132022 PMC4521815

[b12] Bernoulli D. Exposition of a new theory on the measurement of risk. Econometrica: Journal of the Econometric Society, 23–36 (1954).

[b13] Huettel S. A., Stowe C. J., Gordon E. M., Warner B. T. & Platt M. L. Neural signatures of economic preferences for risk and ambiguity. Neuron 49, 765–775 (2006). 16504951 10.1016/j.neuron.2006.01.024

[b14] Hsu M., Bhatt M., Adolphs R., Tranel D. & Camerer C. F. Neural systems responding to degrees of uncertainty in human decision-making. Science 310, 1680–1683 (2005). 16339445 10.1126/science.1115327

[b15] McKelvey R. D. & Palfrey T. R. Quantal Response Equilibria for Normal Form Games. Games and Economic Behavior 10, 6–38 (1995).

[b16] Beck A. T., Steer R. A. & Brown G. K. Manual for the beck depression inventory-II. San Antonio, TX: Psychological Corporation 1, 82 (1996).

[b17] Conover W. J. & Iman R. L. Rank transformations as a bridge between parametric and nonparametric statistics. The American Statistician 35, 124–129 (1981).

[b18] Team J. J. A. S. P. Version 0.8. 0.0. *Computer software* (2016).

[b19] Andersen S., Harrison G. W., Lau M. I. & Elisabet Rutström E. Lost in state space: are preferences stable? International Economic Review 49, 1091–1112 (2008).

[b20] Wölbert E. & Riedl A. Measuring time and risk preferences: Reliability, stability, domain specificity. *CESifo Working Paper Series No. 4339. Available at SSRN:* https://ssrn.com/abstract=2302494 (2013).

[b21] Forbes E. E., Shaw D. S. & Dahl R. E. Alterations in reward-related decision making in boys with recent and future depression. Biological psychiatry 61, 633–639 (2007). 16920074 10.1016/j.biopsych.2006.05.026

[b22] Steele J., Kumar P. & Ebmeier K. P. Blunted response to feedback information in depressive illness. Brain 130, 2367–2374 (2007). 17586866 10.1093/brain/awm150

[b23] Satterthwaite T. D. . Common and dissociable dysfunction of the reward system in bipolar and unipolar depression. Neuropsychopharmacology 40, 2258–2268 (2015). 25767910 10.1038/npp.2015.75 PMC4613620

[b24] Pizzagalli D. A., Jahn A. L. & O’Shea J. P. Toward an objective characterization of an anhedonic phenotype: a signal-detection approach. Biological psychiatry 57, 319–327 (2005). 15705346 10.1016/j.biopsych.2004.11.026 PMC2447922

[b25] Spielberger C. D. & Gorsuch R. L. State-trait anxiety inventory for adults: sampler set: manual, test, scoring key. (Mind Garden, 1983).

[b26] Watson D. . Testing a tripartite model: I. Evaluating the convergent and discriminant validity of anxiety and depression symptom scales. Journal of abnormal psychology 104, 3 (1995). 7897050 10.1037//0021-843x.104.1.3

[b27] Smoski M. J. . Decision-making and risk aversion among depressive adults. Journal of behavior therapy and experimental psychiatry 39, 567–576 (2008). 18342834 10.1016/j.jbtep.2008.01.004 PMC2590786

[b28] Leahy R. L., Tirch D. D. & Melwani P. S. Processes underlying depression: Risk aversion, emotional schemas, and psychological flexibility. International Journal of Cognitive Therapy 5, 362–379 (2012).

[b29] Dichter G. S. . The effects of psychotherapy on neural responses to rewards in major depression. Biological psychiatry 66, 886–897 (2009). 19726030 10.1016/j.biopsych.2009.06.021 PMC3657763

[b30] Forbes E. E. . Reward‐related decision‐making in pediatric major depressive disorder: an fMRI study. Journal of Child Psychology and Psychiatry 47, 1031–1040 (2006). 17073982 10.1111/j.1469-7610.2006.01673.x PMC2129133

[b31] Dulleck U., Fooken J. & Fell J. Within-Subject Intra-and Inter-Method Consistency of Two Experimental Risk Attitude Elicitation Methods. German Economic Review 16, 104–121 (2015).

[b32] Lönnqvist J.-E., Verkasalo M., Walkowitz G. & Wichardt P. C. Measuring individual risk attitudes in the lab: Task or ask? An empirical comparison. Journal of Economic Behavior & Organization 119, 254–266 (2015).

[b33] Roe B. E., Haab T. C., Beversdorf D. Q., Gu H. H. & Tilley M. R. Risk-attitude selection bias in subject pools for experiments involving neuroimaging and blood samples. Journal of Economic Psychology 30, 181–189 (2009).

[b34] Chuang Y. & Schechter L. Stability of experimental and survey measures of risk, time, and social preferences: A review and some new results. Journal of Development Economics 117, 151–170 (2015). 10.1016/j.jdeveco.2015.07.008 PMC6070154 30078930

[b35] Gradin V. B. . Expected value and prediction error abnormalities in depression and schizophrenia. Brain awr059 (2011). 10.1093/brain/awr059 21482548

[b36] Kumar P. . Abnormal temporal difference reward-learning signals in major depression. Brain 131, 2084–2093 (2008). 18579575 10.1093/brain/awn136

[b37] Robinson O. J., Cools R., Carlisi C. O., Sahakian B. J. & Drevets W. C. Ventral striatum response during reward and punishment reversal learning in unmedicated major depressive disorder. American Journal of Psychiatry (2012). 10.1176/appi.ajp.2011.11010137 PMC5648982 22420038

[b38] Chase H. . Approach and avoidance learning in patients with major depression and healthy controls: relation to anhedonia. Psychological medicine 40, 433–440 (2010). 19607754 10.1017/S0033291709990468

[b39] Blanco N. J., Otto A. R., Maddox W. T., Beevers C. G. & Love B. C. The influence of depression symptoms on exploratory decision-making. Cognition 129, 563–568 (2013). 24055832 10.1016/j.cognition.2013.08.018 PMC3809321

[b40] Beevers C. G. . Influence of depression symptoms on history-independent reward and punishment processing. Psychiatry research 207, 53–60 (2013). 23122555 10.1016/j.psychres.2012.09.054 PMC3566413

[b41] Henriques J. B. & Davidson R. J. Decreased responsiveness to reward in depression. Cognition & Emotion 14, 711–724 (2000).

[b42] Hershenberg R. . Diminished effort on a progressive ratio task in both unipolar and bipolar depression. Journal of affective disorders 196, 97–100 (2016). 26919058 10.1016/j.jad.2016.02.003 PMC4808384

[b43] Knutson B., Bhanji J. P., Cooney R. E., Atlas L. Y. & Gotlib I. H. Neural responses to monetary incentives in major depression. Biological psychiatry 63, 686–692 (2008). 17916330 10.1016/j.biopsych.2007.07.023 PMC2290738

[b44] Pizzagalli D. A. . Reduced caudate and nucleus accumbens response to rewards in unmedicated individuals with major depressive disorder. American Journal of Psychiatry 166, 702–710 (2009). 19411368 10.1176/appi.ajp.2008.08081201 PMC2735451

[b45] Richards D. A. . Cost and Outcome of Behavioural Activation versus Cognitive Behavioural Therapy for Depression (COBRA): a randomised, controlled, non-inferiority trial. The Lancet 388, 871–880 (2016). 10.1016/S0140-6736(16)31140-0 PMC5007415 27461440

[b46] First M. B., Spitzer R. L., Gibbon M. & Williams J. B. W. Structured clinical interview for DSM-IV-TR Axis I disorders: Patient edition (with Psychotic Screen) (SCID-I/P (W/ PSYCHOTIC SCREEN), 1/2007 revision). (Biometrics Research Department, New York State Psychiatric Institute, 2007).

[b47] Sheehan D. V. . The Mini-International Neuropsychiatric Interview (MINI): the development and validation of a structured diagnostic psychiatric interview for DSM-IV and ICD-10. Journal of clinical psychiatry (1998). 9881538

[b48] Dozois D. J., Dobson K. S. & Ahnberg J. L. A psychometric evaluation of the Beck Depression Inventory–II. Psychological assessment 10, 83 (1998).

[b49] Daw N. D. Trial-by-trial data analysis using computational models. Decision making, affect, and learning: Attention and performance XXIII 23, 3–38 (2011).

[b50] Niv Y., Edlund J. A., Dayan P. & O’Doherty J. P. Neural prediction errors reveal a risk-sensitive reinforcement-learning process in the human brain. The Journal of Neuroscience 32, 551–562 (2012). 22238090 10.1523/JNEUROSCI.5498-10.2012 PMC6621075

[b51] Storey J. D. A direct approach to false discovery rates. Journal of the Royal Statistical Society: Series B (Statistical Methodology) 64, 479–498 (2002).

