# Supplementary material for: Valuation in major depression is intact and stable in a non-learning environment
Source: Sci Rep. 2017 Mar 10;7:44374. doi: 10.1038/srep44374 (PMC5345037; doi:10.1038/srep44374)
Supplement: Supplementary Information [file srep44374-s1.pdf]

Supplementary Information:

for

Valuation in major depression is intact and stable in a non-learning environment

Dongil Chung,[1] Kelly Kadlec,[1,2,3] Jason A. Aimone,[1,4]
Katherine McCurry,[1,5] Brooks King-Casas,[1,5] Pearl H. Chiu[1,5]

Affiliation:

[1]Virginia Tech Carilion Research Institute, Roanoke, VA
[2]Department of Electrical and Computer Engineering, Virginia Tech, Blacksburg, VA
[3]School of Neuroscience, Virginia Tech, Blacksburg, VA
[4]Department of Economics, Baylor University, TX
[5]Department of Psychology, Virginia Tech, Blacksburg, VA

Supplementary text:

Results from model-free analyses of risk preference

Participants' proportion of risky choices P(risky) in the gambling task was used as a model-free measure of risk preference (per Holt & Laury). This model-free measure captures behavioral trends of choosing the option with larger payoff variance (i.e., the riskier option). Based on the expected value between alternative choices (**Fig. 1**), a risk neutral individual should show P(risky) = 5/9 ≈ 0.56 in the task; higher P(risky) indicates risk seeking behavior.

Participants' proportion of risky choices were calculated for each of four visits to the laboratory, and were compared in the MDD and control groups to examine the stability P(risky) over time. Based on P(risky), both controls and MDD participants showed significant risk aversion (**Supplementary Fig. S2a**; control, Time 1: t(27) = –3.37, *P* = 0.0023; Time 2: t(27) = –4.87, *P* = 4.31e–05; Time 3: t(27) = –3.53, *P* = 0.0015; Time 4: t(27) = –4.74, *P* = 6.19e–05; MDD, T1: t(46) = –5.17, *P* = 5.02e–06; T2: t(46) = –3.43, *P* = 0.0013; T3: t(46) = –3.61, *P* = 7.62e–04; T4: t(46) = –6.10, *P* = 2.06e–07; one-sample t-test). This risk aversion was stable over time. That is, the probability of choosing the risky option did not change across the four visits for either the control or MDD participants (control: F(3, 81) = 0.68, *P* = 0.57, repeated-measures ANOVA; MDD: F(3, 138) = 1.14, *P* =0.34), and were not different between MDD and control groups (F(1,219) = 0.81, *P* = 0.37, mixed-design ANOVA). These results show that MDD patients show comparable risk aversion to controls based on a model-free measure of risk preference, and that behavioral choices were statistically stable across four repeated measures over time.

The stability of P(risky) was also examined by testing whether the rank order of P(risky) was consistent between all 6 pairs of repeated visits (1st visit vs 2nd, 1st vs 3rd, 1st vs 4th, 2nd vs 3rd, 2nd vs 4th, and 3rd vs 4th). Both control and MDD participants showed moderate to high average stability of P(risky) (**Supplementary Fig. S2b**; control: ρ = 0.50; MDD: ρ = 0.37). These results indicate that individuals' risky choice patterns do not change over time.

Model parameter recovery

To address whether the task design is sensitive enough to capture group differences if they existed, we simulated individual choice data (using known mean difference effect sizes and standard deviations for each distribution; 'true parameters' hereafter), and examined whether group differences are recovered or not from the modeling analyses. To test this, we simulated two groups' parameter distributions with *a priori* small, medium, and large effect sizes in the groups' hyperparameters and tested for reproducibility with these simulated data. Each group's risk preference and inverse temperature distributions were defined using lognormal distributions; using a lognormal instead of gamma distribution does not affect group or individual parameter estimation, but is more

straightforward for simulating means and standard deviations. To test if our task design and estimation procedures are sensitive enough to capture group differences (where they exist) at different effect sizes, means of the two groups' parameter distributions were calculated for small, medium, and large *a priori* Cohen's d effect sizes ($mean_{group2}$ = $mean_{group1}$ + effect size × s.t.d.), and standard deviations were fixed as the estimated values from the actual behavioral data pooled across the entire sample of $N = 98$ (0.72 for RP and 0.33 for IT). Specifically, we defined two baseline group mean distributions (for RP and IT, respectively) and six other group distributions to achieve small, medium, and large mean difference effect sizes (Cohen's d = 0.2, 0.5, and 1.0): RP_base ~ lognormal(–1.12, 0.72), RP_s ~ lognormal(–0.98, 0.72), RP_m ~ lognormal(–0.76, 0.72), RP_l ~ lognormal(–0.40, 0.72), IT_base ~ lognormal(1.34, 0.33), IT_s ~ lognormal(1.41, 0.33), IT_m ~ lognormal(1.51, 0.33), and IT_l ~ lognormal(1.67, 0.33). Note that RP_base and IT_base followed the distribution estimates from the actual behavioral data (baseline hereafter). For each test, matching the same sample sizes as the real data, values for 33 pairs of parameters (i.e., RP and IT for 33 simulated subjects) were sampled from the baseline distribution and values for 65 pairs of parameters were sampled from the second group's distribution. After generating the simulated choices, we used the same estimation procedure as reported in the main text to re-estimate (recover) the parameters ('estimated parameters' hereafter).

The first simulated test-set with a small mean difference effect size for RP and IT (Cohen's d = 0.2) did not generate significant mean parameter differences between the sampled groups (RP: $Z = -0.48$, $P = 0.63$; IT: $Z = -1.61$, $P = 0.11$; Wilcoxon rank sum test). This likely reflects the small effect size, in that randomly selected means from sample individuals for each group will not always identify significant group differences. As expected, the re-estimated parameters recovered comparable (non-different) means between the two simulated groups (RP: $Z = -0.40$, $P = 0.68$; IT: $Z = -0.54$, $P = 0.59$; **Fig. S4a**). The second test-set, with medium mean difference effect sizes for RP and IT (Cohen's d = 0.5), had significant mean differences in both RP and IT (RP: $Z = -2.53$, $P = 0.011$; IT: $Z = -2.07$, $P = 0.038$). Applying the modeling analyses reported in the main text to these simulated participants recovered a significant group difference in RP and IT (RP: $Z = -2.01$, $P = 0.045$; IT: $Z = -2.52$, $P = 0.012$; **Fig. S4b**). The third simulated test-set, with large mean difference effect size in RP and IT (Cohen's d = 1.0), had significant group differences for both RP and IT (RP: $Z = -5.16$, $P = 2.51e-07$; IT: $Z = -4.63$, $P = 3.65e-06$), and we were able to recover the true group mean differences in both parameters (RP: $Z = -4.82$, $P = 1.42e-06$; IT: $Z = -3.28$, $P = 0.0010$; **Fig. S4c**). These data thus support the recoverability of the parameters (and parameter differences where they exist).

Note that the individually estimated parameters are positively correlated with the true parameters across the groups regardless of the effect sizes of the group mean differences (baseline vs. small, RP: spearman's correlation $\rho = 0.76$, $P = 5.57e-20$, IT: $\rho = 0.34$, $P = 7.18e-04$; baseline vs. medium, RP: $\rho = 0.69$, $P = 2.79e-15$, IT: $\rho = 0.42$, $P = 1.98e-05$; baseline vs. large, RP: $\rho = 0.88$, $P = 1.38e-33$, IT: $\rho = 0.20$, $P = 0.050$). This further supports the recoverability of the parameters (and parameter differences if they existed).

Goodness-of-fit of the model compared with alternative models

To test the goodness of fit of the reported (target) two-parameter model including RP and IT, we compared the model's estimation performance against four alternative models, including: i) a random choice model, where all choices are at chance level, ii) an inverse temperature only model, where risk neutrality is assumed (RP = 1) for all participants, iii) a risk preference only model, in which the same level of IT (a group-level parameter was used) is applied to all participants, and iv) a three-parameter model, where in addition to RP and IT, a third parameter explains nonlinear transformation of probabilities. Specifically for the nonlinear transformation of probabilities, we used the one-parameter probability weighting function (PWF) from Prelec (1998). We estimated parameters for each model and measured model fit using Bayesian Information Criteria (BIC) which takes into account the number of parameters in each model (lower values indicate better model fit). Specifically, we first calculated the likelihood of individual participants' behavioral choices given the set of parameters for each model that maximized the posterior (from a maximum-a-posteriori (MAP) estimation). Then for each model, the number of free parameters (e.g., 2 for the model that includes risk preference and inverse temperature) was taken into account for computing BIC. The computed BIC scores were summed across subjects and visits. Among the tested models, the two-parameter and three-parameter models showed comparably good model fits compared with other three alternative models ($BIC_{\text{Random choice}} = 4891$, $BIC_{IT} = 4013$, $BIC_{RP} = 4260$, $BIC_{RP+IT} = 3990$, and $BIC_{RP+IT+PWF} = 3982$). Although the three-parameter model showed a comparable model fit with the target model, the three-parameter model's parameters could not be recovered. These results show that the three-parameter model may be too complex for the present task design. We believe that a future study including finer increments of probability and an increased number of trials may address whether nonlinear probability perceptions exist in individuals with MDD.

Two additional approaches for parameter estimation

As an approach with less bias against or toward finding group differences within these empirical Bayes methods, we introduced an additional variable for capturing potential group mean differences i) in RP or ii) in IT. This variable ΔRP (or ΔIT) was used to represent a between-group difference in RP (or IT), while still allowing each group to have separate hyperparameters. Specifically, applying the same assumption as above of hierarchical structure, we hypothesized that RP (or IT) of individuals in both control and MDD groups are samples from the same group-level distribution. One critical feature here is that for testing between group differences, ΔRP (or ΔIT), a group-level parameter, was added to all individuals with MDD. That is, ΔRP allows the mean of the group-level distribution of RP to be different (or not) between the MDD and the control groups; ΔRP = 0 indicates that the means of the two groups' group-level distributions are the same, and ΔRP different from zero indicates that the two groups have different group-level means for their distributions.

Because ΔRP is defined as a group-level mean difference (single value point estimation using maximum likelihood estimation), we took a jackknife approach to calculate the parameter's standard deviation and to make an inference about whether the estimated difference is different from zero or not; 65 group-level estimations were conducted, leaving one participant out for each of the ΔRP estimation (65 individuals with MDD). Estimated results show that neither ΔRT nor ΔIT is different from zero (ΔRT, mean = –0.094, s.e. = 0.053, 95% confidence interval = [–0.20, 0.010]; ΔIT, mean = –1.49, s.e. = 0.89, CI = [–3.23, 0.24]). This provides additional evidence showing that individuals with MDD do not have different RP or IT from control individuals.

An approach biasing against finding NO group differences is to use a separate prior for each group (opposite from our original 'equal prior' approach). Compared with the two previous approaches, using separate priors is more likely to find group differences if there are any existing group differences. We also re-estimated the data using this 'separate prior' approach and identified results comparable with what we reported in the manuscript. Specifically, there were no significant main effects of group and repeated visits, and no significant interaction of group x visit for either RP or IT (RP, Group: $F(1, 219) = 3.37$, $P = 0.071$; RP, Visit: $F(3, 219) = 0.82$, $P = 0.49$; RP, Group x Visit: $F(3, 219) = 0.48$, $P = 0.70$; IT, Group: $F(1, 219) = 1.43$, $P = 0.24$; IT, Visit: $F(3, 219) = 1.48$, $P = 0.22$; IT, Group x Visit: $F(3, 219) = 0.77$, $P = 0.51$).

Null hypothesis significance testing of value sensitivities between groups and across visits

Given that the tested effects of the group difference and repeated visits were not significant, we examined the strength of evidence for the null hypotheses using null hypothesis significance testing. Consistent with the statistical results using conventional frequentist approaches, the Bayes factors (BF) showed supporting evidence for the null hypothesis against alternative models (**Table S5**). Particularly, both RP and IT showed very strong evidence for null hypothesis against the full model that includes both main effects of group and repeated visit, and their interaction effect ($BF_{01} > 30$). Against an alternative model that only included the main effect of group, both parameters also showed evidence for the null hypothesis ($1 < BF_{01} < 3$); these results are consistent with the originally reported null results using non-Bayesian hypothesis testing.

Potential practice effect across visits

There is a possibility of practice effects in the repeated-measures design. If there were any practice effects, these should be captured as changes correlated with time. As reported in the main text, RP and IT were stable across visits, indicating no temporal change in healthy controls or individuals with MDD (**Fig. 2bi**). To directly examine whether there are changes as a function of repeated visits, we measured the slope of changes in each individual's RP and IT across four visits (linear regression). **Figure S5** indicates that healthy controls and individuals with MDD show a mean slope for RP and

IT comparable to zero (Wilcoxon's signed rank test; $RP_{control}$: Z = 1.25, *P* = 0.21; RP_{MDD}: Z = 0.12, *P* = 0.91; $IT_{control}$: Z = 1.87, *P* = 0.062; IT_{MDD}: Z = 0.94, *P* = 0.35). Although healthy controls showed a marginally positive mean slope for IT as a group, the average slopes were not different between the groups (Wilcoxon's rank sum test, Z = 1.19, *P* = 0.23). These results indicate that there are no significant practice effects across repeated visits. Given the marginally significant trend of increased IT across visits within the healthy control group, future work using a larger number of repetitions may examine the possibility of practice effects and changes over time.

Supplementary Tables

Table S1. Demographic and symptom data for individuals with major depression who received cognitive behavioral therapy

	Major depression with treatment (*N* = 45)
Male/female participants	10/35
Age (years)	39.69 ± 11.92
Verbal intelligence quotient[a]	107.82 ± 11.85
BDI-II	
Time 1	30.62 ± 7.08 (45)
Time 2	22.16 ± 10.55 (45)
Time 3	16.81 ± 11.95 (42)
Time 4	13.07 ± 12.34 (44)
BDI, Anhedonia subscale[b]	
Time 1	49.11 ± 10.72 (45)
Time 2	46.47 ± 11.61 (45)
Time 3	41.50 ± 12.40 (44)
Time 4	36.76 ± 11.63 (45)
State Anxiety	
Time 1	49.11 ± 10.72 (45)
Time 2	46.47 ± 11.61 (45)
Time 3	41.50 ± 12.40 (44)
Time 4	36.76 ± 11.63 (45)
MASQ subscales	
Anhedonic Depression	
Time 1	82.71 ± 8.42 (45)
Time 2	70.91 ± 14.54 (44)
Time 3	64.20 ± 18.89 (44)
Time 4	61.00 ± 17.21 (45)
Anxious Arousal	
Time 1	26.00 ± 7.00 (45)
Time 2	24.23 ± 7.12 (44)
Time 3	22.91 ± 7.90 (44)
Time 4	21.87 ± 6.35 (45)
GD:Anxiety	
Time 1	25.33 ± 7.18 (45)
Time 2	21.05 ± 6.32 (44)
Time 3	19.61 ± 7.44 (44)
Time 4	17.33 ± 5.53 (45)
GD: Depression	

Time 1	39.07 ± 8.56 (45)
Time 2	30.11 ± 9.16 (44)
Time 3	25.77 ± 10.45 (44)
Time 4	23.53 ± 10.44 (45)
GD: Mixed	
Time 1	45.27 ± 8.84 (45)
Time 2	38.36 ± 9.16 (44)
Time 3	35.09 ± 10.56 (44)
Time 4	31.13 ± 10.28 (45)

BDI-II, Beck Depression Inventory, Second Edition; State Anxiety, State Anxiety Scale of the State-Trait Anxiety Inventory; MASQ, Mood and Anxiety Symptom Questionnaire; GD, General Distress; [a]Verbal intelligence quotient scores were measured with the Wechsler Test of Adult Reading (WTAR); [b]The Anhedonia subscale was created by summing responses on the following BDI-II items associated with anhedonia symptoms: loss of pleasure (item 4), loss of interest (item 12), loss of energy (item 15), and loss of interest in sex (item 21). Numbers of participants who were included for calculating mean and standard deviation of each questionnaire score are noted in parentheses; see **Supplementary Fig. S1** for the BDI-II, STAI, and MASQ scores in the smallest subset of included participants (visiting all four times), and see **Methods** for details about inclusion criteria.

Table S2. Spearman correlation coefficients of value sensitivities between visits to the laboratory (([1st vs 2nd visit], [1st vs 3rd visit], … [3rd vs 4th visit]).

	Control	Major depression
Risk preference		
Time 1 vs Time 2	ρ = 0.52, P = 0.0028 (31)	ρ = 0.54, P = 2.84e-05 (54)
Time 1 vs Time 3	ρ = 0.63, P = 2.57e-04 (29)	ρ = 0.41, P = 0.0025 (51)
Time 1 vs Time 4	ρ = 0.65, P = 3.72e-05 (33)	ρ = 0.52, P = 8.26e-06 (65)
Time 2 vs Time 3	ρ = 0.32, P = 0.092 (28)	ρ = 0.58, P = 1.84e-05 (47)
Time 2 vs Time 4	ρ = 0.68, P = 2.91e-05 (31)	ρ = 0.55, P = 1.61e-05 (54)
Time 3 vs Time 4	ρ = 0.59, P = 7.22e-04 (29)	ρ = 0.65, P = 2.87e-07 (51)
Inverse temperature		
Time 1 vs Time 2	ρ = 0.30, P = 0.10 (31)	ρ = 0.54, P = 2.74e-05 (54)
Time 1 vs Time 3	ρ = 0.52, P = 0.0039 (29)	ρ = 0.48, P = 3.26e-04 (51)
Time 1 vs Time 4	ρ = 0.32, P = 0.071 (33)	ρ = 0.54, P = 2.80e-06 (65)
Time 2 vs Time 3	ρ = 0.56, P = 0.0017 (28)	ρ = 0.63, P = 1.98e-06 (47)
Time 2 vs Time 4	ρ = 0.42, P = 0.020 (31)	ρ = 0.59, P = 2.40e-06 (54)
Time 3 vs Time 4	ρ = 0.76, P = 2.18e-06 (29)	ρ = 0.64, P = 4.44e-07 (51)
Model-free risk preference		
Time 1 vs Time 2	ρ = 0.48, P = 0.0060 (31)	ρ = 0.33, P = 0.015 (54)
Time 1 vs Time 3	ρ = 0.36, P = 0.058 (29)	ρ = 0.37, P = 0.0084 (51)
Time 1 vs Time 4	ρ = 0.55, P = 8.10e-04 (33)	ρ = 0.23, P = 0.061 (65)
Time 2 vs Time 3	ρ = 0.61, P = 5.53e-04 (28)	ρ = 0.44, P = 0.0018 (47)
Time 2 vs Time 4	ρ = 0.62, P =1.86e-04 (31)	ρ = 0.34, P = 0.013 (54)
Time 3 vs Time 4	ρ = 0.41, P = 0.029 (29)	ρ = 0.51, P = 1.29e-04 (51)

Numbers of participants who were included for calculating mean and standard deviation of each questionnaire score are noted in parentheses. All correlations were significant after applying multiple comparison correction (FDR $q < 0.005$).

Table S3. Spearman correlation coefficients between participants' value sensitivities and symptom severity at each laboratory visit

	Control	Major depression
BDI-II vs.		
Inverse temperature		
Time 1	ρ = 0.13, P = 0.46	ρ = –0.15, P = 0.25
Time 2	ρ = –0.11, P = 0.56	ρ = –0.17, P = 0.24
Time 3	ρ = 0.073, P = 0.71	ρ = –0.19, P = 0.19
Time 4	ρ = 0.35, P = 0.043	ρ = –0.30, P = 0.016
Risk preference		
Time 1	ρ = –0.13, P = 0.48	ρ = 0.082, P = 0.52
Time 2	ρ = –0.16, P = 0.39	ρ = –0.21, P = 0.14
Time 3	ρ = –0.34, P = 0.073	ρ = –0.15, P = 0.32
Time 4	ρ = 0.060, P = 0.74	ρ = –0.12, P = 0.34
BDI, Anhedonia subscale vs.		
Inverse temperature		
Time 1	ρ = 0.090, P = 0.62	ρ = –0.14, P = 0.28
Time 2	ρ = –0.23, P = 0.21	ρ = –0.19, P = 0.18
Time 3	ρ = –0.053, P = 0.78	ρ = –0.19, P = 0.19
Time 4	ρ = 0.35, P = 0.043	ρ = –0.25, P = 0.049
Risk preference		
Time 1	ρ = –0.21, P = 0.23	ρ = –0.047, P = 0.71
Time 2	ρ = –0.38, P = 0.036	ρ = –0.28, P = 0.042
Time 3	ρ = –0.32, P = 0.087	ρ = –0.16, P = 0.25
Time 4	ρ = 0.080, P = 0.66	ρ = –0.081, P = 0.52
State Anxiety vs.		
Inverse temperature		
Time 1	ρ = 0.25, P = 0.16	ρ = 0.020, P = 0.88
Time 2	ρ = 0.072, P = 0.70	ρ = 0.0001, P = 1.00
Time 3	ρ = 0.091, P = 0.64	ρ = –0.084, P = 0.56
Time 4	ρ = 0.19, P = 0.28	ρ = –0.17, P = 0.18
Risk preference		
Time 1	ρ = 0.035, P = 0.85	ρ = 0.038, P = 0.76
Time 2	ρ = 0.19, P = 0.31	ρ = –0.041, P = 0.77
Time 3	ρ = –0.32, P = 0.090	ρ = –0.022, P = 0.88
Time 4	ρ = 0.19, P = 0.29	ρ = 0.049, P = 0.70
MASQ subscales		
Anhedonic depression vs.		
Inverse temperature		
Time 1	ρ = 0.31, P = 0.081	ρ = –0.11, P = 0.37
Time 2	ρ = 0.29, P = 0.11	ρ = 0.095, P = 0.51
Time 3	ρ = 0.18, P = 0.36	ρ = –0.18, P = 0.21
Time 4	ρ = 0.096, P = 0.60	ρ = –0.16, P = 0.21
Risk preference		
Time 1	ρ = 0.11, P = 0.53	ρ = 0.059, P = 0.64
Time 2	ρ = 0.31, P = 0.091	ρ = 0.029, P = 0.84

Time 3	ρ = –0.23, P = 0.23	ρ = –0.070, P = 0.63
Time 4	ρ = –0.084, P = 0.65	ρ = –0.078, P = 0.54
Anxious arousal vs.		
Inverse temperature		
Time 1	ρ = –0.085, P = 0.64	ρ = –0.12, P = 0.33
Time 2	ρ = 0.17, P = 0.35	ρ = –0.23, P = 0.099
Time 3	ρ = –0.084, P = 0.67	ρ = –0.31, P = 0.031
Time 4	ρ = 0.15, P = 0.43	ρ = –0.13, P = 0.31
Risk preference		
Time 1	ρ = –0.22, P = 0.23	ρ = –0.0032, P = 0.98
Time 2	ρ = –0.21, P = 0.26	ρ = –0.14, P = 0.33
Time 3	ρ = –0.35, P = 0.062	ρ = –0.15, P = 0.31
Time 4	ρ = 0.13, P = 0.48	ρ = –0.14, P = 0.26
GD: Anxiety vs.		
Inverse temperature		
Time 1	ρ = 0.029, P = 0.87	ρ = –0.17, P = 0.17
Time 2	ρ = 0.045, P = 0.81	ρ = –0.18, P = 0.21
Time 3	ρ = 0.10, P = 0.59	ρ = –0.038, P = 0.79
Time 4	ρ = 0.18, P = 0.32	ρ = –0.19, P = 0.14
Risk preference		
Time 1	ρ = –0.11, P = 0.54	ρ = 0.0049, P = 0.97
Time 2	ρ = 0.012, P = 0.95	ρ = –0.065, P = 0.65
Time 3	ρ = –0.19, P = 0.32	ρ = –0.0019, P = 0.99
Time 4	ρ = 0.28, P = 0.13	ρ = –0.077, P = 0.54
GD: Depression vs.		
Inverse temperature		
Time 1	ρ = –0.13, P = 0.47	ρ = –0.032, P = 0.80
Time 2	ρ = 0.28, P = 0.13	ρ = –0.12, P = 0.40
Time 3	ρ = 0.16, P = 0.41	ρ = –0.20, P = 0.17
Time 4	ρ = 0.20, P = 0.28	ρ = –0.15, P = 0.22
Risk preference		
Time 1	ρ = –0.28, P = 0.12	ρ = 0.26, P = 0.034
Time 2	ρ = 0.12, P = 0.54	ρ = –0.21, P = 0.15
Time 3	ρ = –0.41, P = 0.026	ρ = –0.25, P = 0.075
Time 4	ρ = –0.22, P = 0.22	ρ = –0.063, P = 0.62
GD: Mixed vs.		
Inverse temperature		
Time 1	ρ = 0.028, P = 0.88	ρ = –0.028, P = 0.83
Time 2	ρ = 0.20, P = 0.28	ρ = –0.27, P = 0.052
Time 3	ρ = 0.14, P = 0.46	ρ = –0.23, P = 0.11
Time 4	ρ = 0.14, P = 0.46	ρ = –0.22, P = 0.085
Risk preference		
Time 1	ρ = –0.16, P = 0.37	ρ = 0.16, P = 0.20
Time 2	ρ = 0.18, P = 0.32	ρ = –0.21, P = 0.14
Time 3	ρ = –0.18, P = 0.35	ρ = –0.086, P = 0.55
Time 4	ρ = –0.26, P = 0.16	ρ = –0.030, P = 0.81

BDI-II, Beck Depression Inventory, Second Edition; State Anxiety, State Anxiety Scale of the State-Trait Anxiety Inventory; MASQ, Mood and Anxiety Symptom Questionnaire; GD, General Distress; None of the correlations were significant after applying multiple comparison correction (FDR $q > 0.20$).

Table S4. Spearman correlations between changes in participants' value sensitivities and changes in symptom severity between laboratory visits

	Control	Major depression
ΔBDI-II vs.		
ΔInverse temperature		
T1 – T2	ρ = 0.28, P = 0.14	ρ = –0.18, P = 0.19
T1 – T3	ρ = 0.21, P = 0.29	ρ = –0.033, P = 0.82
T1 – T4	ρ = 0.20, P = 0.27	ρ = –0.026, P = 0.84
T2 – T3	ρ = 0.054, P = 0.79	ρ = –0.032, P = 0.84
T2 – T4	ρ = 0.21, P = 0.27	ρ = –0.13, P = 0.37
T3 – T4	ρ = 0.35, P = 0.060	ρ = –0.36, P = 0.014
ΔRisk preference		
T1 – T2	ρ = 0.13, P = 0.50	ρ = –0.15, P = 0.27
T1 – T3	ρ = –0.15, P = 0.43	ρ = –0.098, P = 0.51
T1 – T4	ρ = 0.11, P = 0.55	ρ = –0.091, P = 0.47
T2 – T3	ρ = –0.12, P = 0.54	ρ = –0.17, P = 0.29
T2 – T4	ρ = 0.25, P = 0.18	ρ = –0.045, P = 0.75
T3 – T4	ρ = –0.10, P = 0.60	ρ = 0.0045, P = 0.98
ΔBDI, Anhedonia subscale vs.		
ΔInverse temperature		
T1 – T2	ρ = 0.25, P = 0.18	ρ = 0.045, P = 0.75
T1 – T3	ρ = 0.26, P = 0.18	ρ = –0.021, P = 0.88
T1 – T4	ρ = 0.28, P = 0.12	ρ = 0.013, P = 0.92
T2 – T3	ρ = –0.061, P = 0.76	ρ = –0.032, P = 0.83
T2 – T4	ρ = –0.025, P = 0.90	ρ = 0.037, P = 0.80
T3 – T4	ρ = 0.26, P = 0.17	ρ = –0.21, P = 0.14
ΔRisk preference		
T1 – T2	ρ = –0.039, P = 0.83	ρ = –0.094, P = 0.51
T1 – T3	ρ = –0.34, P = 0.068	ρ = 0.019, P = 0.89
T1 – T4	ρ = 0.055, P = 0.76	ρ = –0.11, P = 0.39
T2 – T3	ρ = –0.073, P = 0.71	ρ = –0.10, P = 0.51
T2 – T4	ρ = 0.18, P = 0.34	ρ = –0.032, P = 0.82
T3 – T4	ρ = 0.17, P = 0.38	ρ = 0.051, P = 0.72
ΔState Anxiety vs.		
ΔInverse temperature		
T1 – T2	ρ = 0.30, P = 0.10	ρ = 0.075, P = 0.60
T1 – T3	ρ = 0.19, P = 0.32	ρ = –0.032, P = 0.82
T1 – T4	ρ = 0.23, P = 0.20	ρ = 0.11, P = 0.39
T2 – T3	ρ = –0.37, P = 0.050	ρ = 0.030, P = 0.85
T2 – T4	ρ = –0.081, P = 0.66	ρ = –0.075, P = 0.60
T3 – T4	ρ = –0.060, P = 0.76	ρ = –0.087, P = 0.55
ΔRisk preference		
T1 – T2	ρ = –0.060, P = 0.75	ρ = 0.0031, P = 0.98
T1 – T3	ρ = –0.082, P = 0.67	ρ = –0.021, P = 0.89
T1 – T4	ρ = 0.028, P = 0.88	ρ = 0.033, P = 0.80

T2 – T3	ρ = –0.14, P = 0.47	ρ = –0.14, P = 0.37
T2 – T4	ρ = –0.030, P = 0.87	ρ = –0.031, P = 0.83
T3 – T4	ρ = –0.16, P = 0.42	ρ = 0.087, P = 0.55
MASQ subscales		
ΔAnhedonic depression vs.		
ΔInverse temperature		
T1 – T2	ρ = 0.49, P = 0.0054	ρ = –0.092, P = 0.52
T1 – T3	ρ = 0.21, P = 0.27	ρ = –0.12, P = 0.41
T1 – T4	ρ = 0.22, P = 0.24	ρ = 0.015, P = 0.91
T2 – T3	ρ = –0.24, P = 0.23	ρ = –0.23, P = 0.14
T2 – T4	ρ = 0.087, P = 0.65	ρ = –0.012, P = 0.93
T3 – T4	ρ = –0.029, P = 0.88	ρ = –0.24, P = 0.099
ΔRisk preference		
T1 – T2	ρ = 0.068, P = 0.72	ρ = 0.085, P = 0.55
T1 – T3	ρ = 0.086, P = 0.66	ρ = –0.0099, P = 0.95
T1 – T4	ρ = –0.043, P = 0.82	ρ = –0.042, P = 0.74
T2 – T3	ρ = 0.25, P = 0.20	ρ = –0.085, P = 0.58
T2 – T4	ρ = –0.20, P = 0.29	ρ = –0.090, P = 0.53
T3 – T4	ρ = 0.064, P = 0.75	ρ = –0.10, P = 0.47
ΔAnxious arousal vs.		
ΔInverse temperature		
T1 – T2	ρ = 0.098, P = 0.60	ρ = –0.086, P = 0.55
T1 – T3	ρ = –0.17, P = 0.38	ρ = 0.11, P = 0.48
T1 – T4	ρ = –0.077, P = 0.67	ρ = –0.029, P = 0.82
T2 – T3	ρ = –0.22, P = 0.25	ρ = 0.024, P = 0.88
T2 – T4	ρ = –0.13, P = 0.48	ρ = –0.035, P = 0.81
T3 – T4	ρ = –0.30, P = 0.13	ρ = –0.12, P = 0.39
ΔRisk preference		
T1 – T2	ρ = –0.15, P = 0.42	ρ = –0.0076, P = 0.96
T1 – T3	ρ = –0.29, P = 0.13	ρ = –0.0076, P = 0.96
T1 – T4	ρ = –0.27, P = 0.14	ρ = –0.15, P = 0.23
T2 – T3	ρ = –0.22, P = 0.26	ρ = –0.0095, P = 0.95
T2 – T4	ρ = –0.25, P = 0.18	ρ = –0.044, P = 0.76
T3 – T4	ρ = –0.18, P = 0.37	ρ = 0.17, P = 0.22
ΔGD: Anxiety vs.		
ΔInverse temperature		
T1 – T2	ρ = 0.013, P = 0.95	ρ = –0.061, P = 0.67
T1 – T3	ρ = –0.30, P = 0.11	ρ = 0.052, P = 0.72
T1 – T4	ρ = –0.16, P = 0.38	ρ = –0.041, P = 0.75
T2 – T3	ρ = –0.31, P = 0.11	ρ = –0.11, P = 0.47
T2 – T4	ρ = –0.041, P = 0.83	ρ = –0.12, P = 0.39
T3 – T4	ρ = 0.051, P = 0.80	ρ = –0.26, P = 0.068
ΔRisk preference		
T1 – T2	ρ = –0.039, P = 0.84	ρ = –0.013, P = 0.93
T1 – T3	ρ = –0.46, P = 0.012	ρ = 0.14, P = 0.32
T1 – T4	ρ = –0.49, P = 0.0041	ρ = 0.13, P = 0.30

T2 – T3	ρ = –0.25, P = 0.20	ρ = 0.017, P = 0.91
T2 – T4	ρ = –0.096, P = 0.61	ρ = 0.21, P = 0.15
T3 – T4	ρ = –0.16, P = 0.42	ρ = 0.31, P = 0.028
ΔGD: Depression vs.		
ΔInverse temperature		
T1 – T2	ρ = 0.31, P = 0.091	ρ = –0.16, P = 0.27
T1 – T3	ρ = 0.092, P = 0.63	ρ = –0.045, P = 0.76
T1 – T4	ρ = –0.19, P = 0.30	ρ = –0.0055, P = 0.97
T2 – T3	ρ = 0.26, P = 0.19	ρ = –0.11, P = 0.47
T2 – T4	ρ = –0.016, P = 0.93	ρ = –0.16, P = 0.26
T3 – T4	ρ = 0.25, P = 0.20	ρ = –0.35, P = 0.013
ΔRisk preference		
T1 – T2	ρ = –0.14, P = 0.44	ρ = 0.0036, P = 0.98
T1 – T3	ρ = –0.36, P = 0.058	ρ = 0.043, P = 0.77
T1 – T4	ρ = –0.12, P = 0.53	ρ = 0.051, P = 0.69
T2 – T3	ρ = 0.20, P = 0.31	ρ = –0.069, P = 0.66
T2 – T4	ρ = –0.31, P = 0.093	ρ = –0.039, P = 0.79
T3 – T4	ρ = 0.17, P = 0.39	ρ = –0.056, P = 0.70
ΔGD: Mixed vs.		
ΔInverse temperature		
T1 – T2	ρ = 0.24, P = 0.19	ρ = –0.10, P = 0.48
T1 – T3	ρ = –0.054, P = 0.78	ρ = 0.13, P = 0.38
T1 – T4	ρ = –0.032, P = 0.86	ρ = 0.036, P = 0.78
T2 – T3	ρ = –0.066, P = 0.74	ρ = –0.066, P = 0.67
T2 – T4	ρ = 0.10, P = 0.59	ρ = –0.13, P = 0.36
T3 – T4	ρ = –0.37, P = 0.050	ρ = –0.23, P = 0.10
ΔRisk preference		
T1 – T2	ρ = –0.19, P = 0.31	ρ = 0.037, P = 0.80
T1 – T3	ρ = –0.14, P = 0.46	ρ = 0.034, P = 0.81
T1 – T4	ρ = –0.29, P = 0.11	ρ = –0.056, P = 0.66
T2 – T3	ρ = –0.080, P = 0.69	ρ = –0.069, P = 0.66
T2 – T4	ρ = –0.27, P = 0.15	ρ = –0.061, P = 0.67
T3 – T4	ρ = –0.13, P = 0.52	ρ = 0.099, P = 0.50

BDI-II, Beck Depression Inventory, Second Edition; State Anxiety, State Anxiety Scale of the State-Trait Anxiety Inventory; MASQ, Mood and Anxiety Symptom Questionnaire; GD, General Distress; 'T_i – T_j' indicates the difference in each measure between 'Time i' and 'Time j' (i ≠ j); None of the correlations were significant after applying multiple comparison correction (FDR q > 0.39).

Table S5. Null hypothesis significance testing results using Bayesian repeated measures ANOVA. Bayes factors show evidence for the null hypothesis against all potential alternative models.

Models	BF_{01}
Risk preference	
Null model (including subject)	1.00
Group	2.83
Visits	15.65
Group + Visits	43.26
Group + Visits + Group*Visits	433.15
Inverse temperature	
Null model (including subject)	1.00
Group	1.42
Visits	13.03
Group + Visits	18.41
Group + Visits + Group*Visits	98.85

Supplementary Figures and Legends

Figure S1:

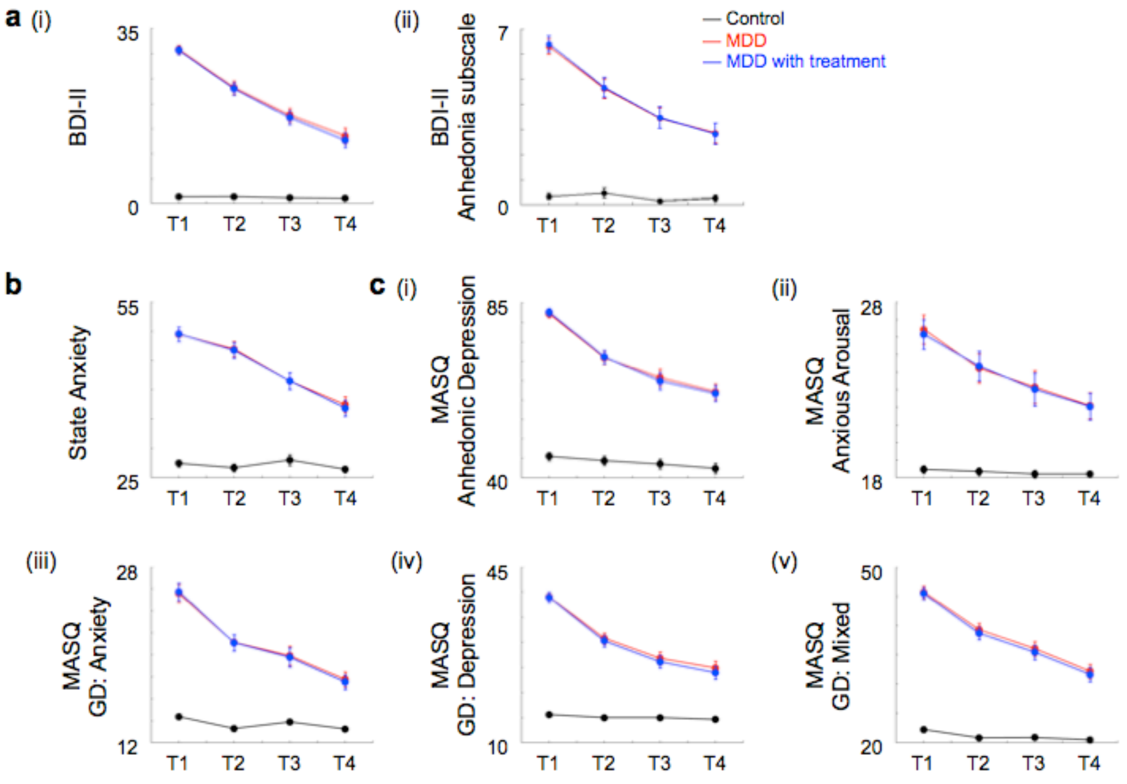

Figure S1. Clinical symptom measures in healthy controls (black) and individuals with MDD (red) across four laboratory visits. As part of a larger ongoing study, a subgroup of individuals with MDD received cognitive behavioral therapy over the course of participation (blue). Only the participants whose questionnaire measures from all four visits exist were included in these plots. BDI-II, Beck Depression Inventory, Second Edition; State Anxiety, State Anxiety Scale of the State-Trait Anxiety Inventory; MASQ, Mood and Anxiety Symptom Questionnaire; GD: General Distress; error bars represent s.e.m.

Figure S2:

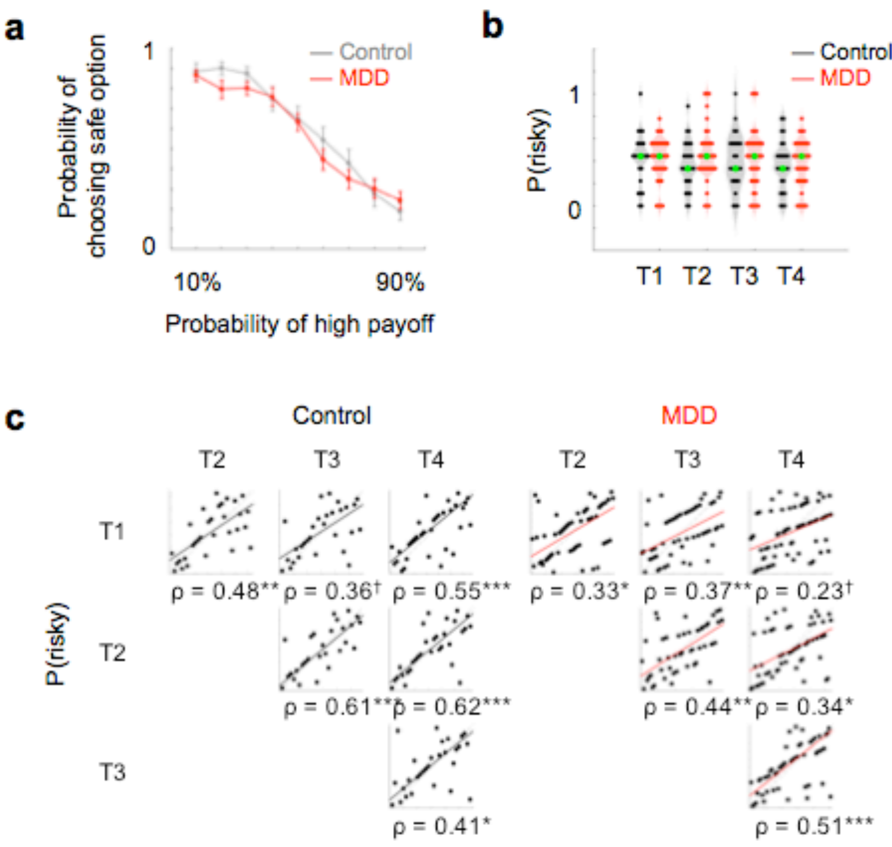

Figure S2. Model-free risk preference is comparable between non-psychiatric controls and MDD and stable across visits. Participants' choices from the gamble task were measured at each visit. **(a)** Pooling the behavioral choices across four visits, healthy controls and individuals with MDD showed comparable choice patterns across high payoff probability. Error bars represent s.e.m. **(b)** As a model-free measure of risk preference, the proportion of risky choices was used. Both groups showed comparable levels of risk aversion across repeated visits, and model-free risk preferences were not different between groups at any time point. Each point represents an individual participant; group medians are indicated in green. Gray and red shades show the distribution of data points along the y-axis. **(c)** Spearman's correlation coefficients were calculated to test whether the rank order of P(risky) among individuals was consistent between visits to the lab (([1st vs 2nd visit], [1st vs 3rd visit], … [3rd vs 4th visit])). See **Supplementary Table S2** for statistical results. Error bars represent s.e.m; each point is an individual participant, and the color-coded lines are the robust regression line between measures from two visits. The x- and y-axes each represent the rank order of individual participants at each visit (for simplicity, not labeled here); $^{\dagger}P < 0.07$, $*P < 0.05$, $**P < 0.01$, $***P < 0.001$, uncorrected; all correlations were significant after applying multiple comparison correction (FDR $q < 0.005$).

Figure S3:

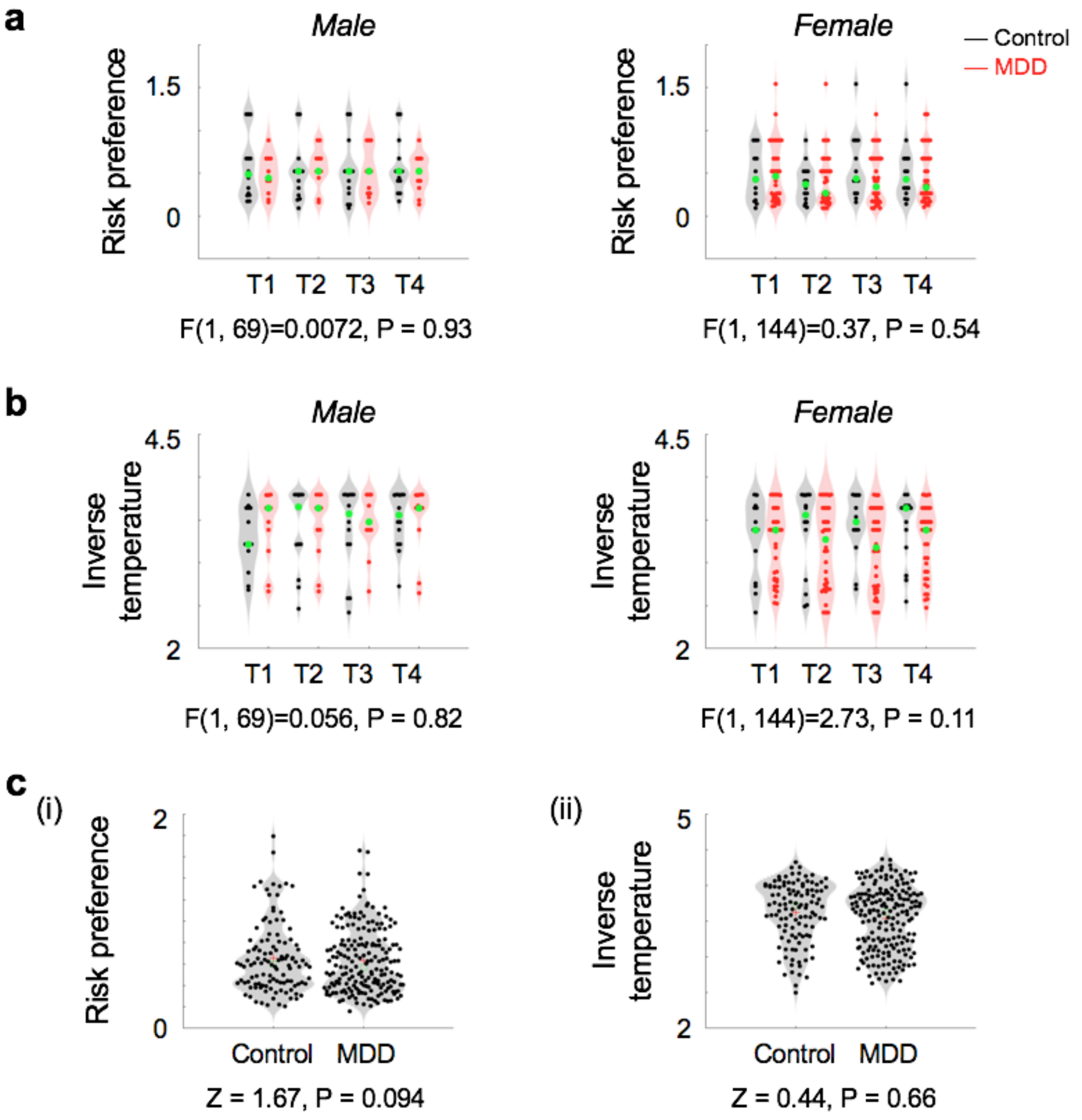

Figure S3. Risk preference and inverse temperature estimates are not different between genders, and are stable for both genders across visits; risk preference and inverse temperature are comparable between groups after controlling for age. (a,b) mixed-design ANOVA was used for statistical analyses after rank transformation. Each point represents an individual participant, and each green dot represents a group median. Gray and red shades represent distribution of data points along the y-axis. **(c)** To control for age effects, we used linear regression where age was an independent variable, and risk preference and inverse temperature were dependent variables. The residuals from each regression analyses and Wilcoxon rank-sum test were used for between-group comparison in each parameter. Error bars represent s.e.m.; each point represents an individual participant; gray shades represent distribution of data points along the y-axis.

Figure S4:

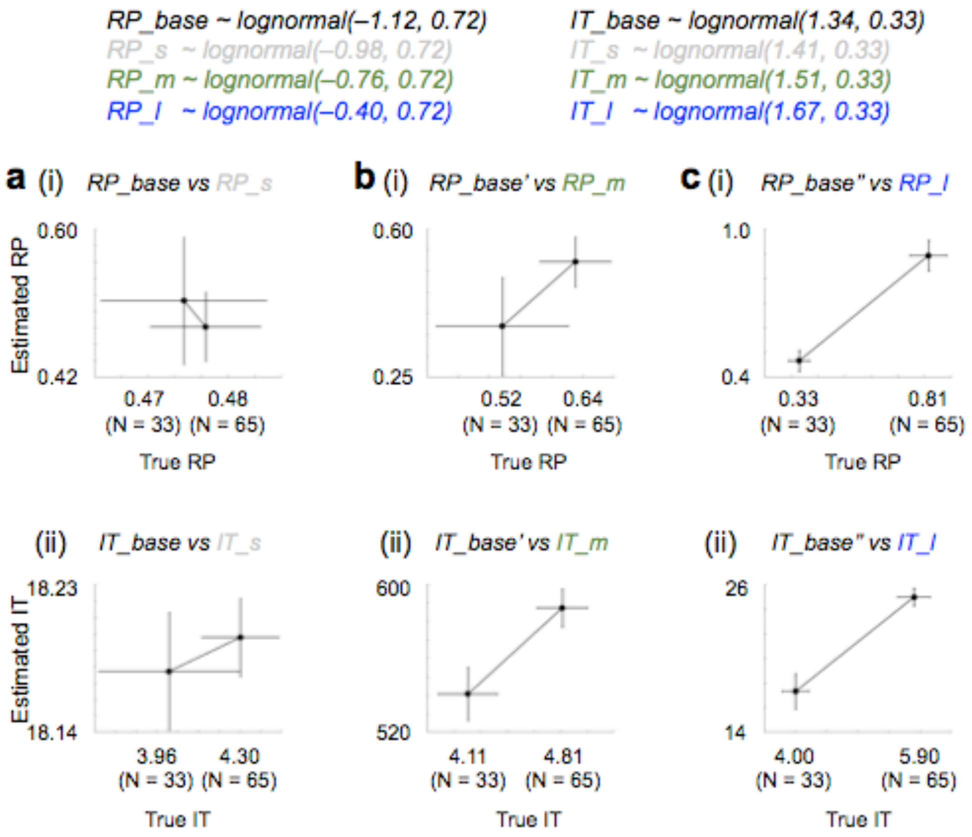

Figure S4. Model parameter recovery from simulated choice data. Model parameter recovery from simulated choice data. To test if our task design and estimation procedures are sensitive enough to capture group differences (where they exist) at different effect sizes, means generating **(a)** small, **(b)** medium, and **(c)** large mean difference effect sizes (Cohen's d = 0.2, 0.5, and 1.0) were simulated for each parameter, and standard deviations were fixed as the estimated values from the actual behavioral data of the entire sample (0.72 for RP and 0.33 for IT). We sampled 33 pairs of parameters as the first simulated group (RP and IT for 33 simulated subjects) from lognormal distributions where their mean and standard deviations are known. In addition, we sampled 65 pairs of parameters from lognormal distributions where the parameters' reflected small (_s), medium (_m), and large (_l) mean difference effect sizes compared with the first simulated group (_base). Nine choices were simulated for each pair of these 'true parameters' (x-axes) and used for re-estimating the parameters (estimated parameters; y-axes). Values along the x-axes are mean parameter values for each simulated group. The first simulated test-set (small mean difference effect size) did not generate significant mean parameter differences and the re-estimated parameters similarly were not different. The mean differences that were generated in the second and third test-sets (medium, large mean difference effect sizes) were recovered from the parameter estimation. These results indicate that the model is sensitive enough to detect the group differences in both RP and IT should they exist. Error bars represent s.e.m.

Figure S5:

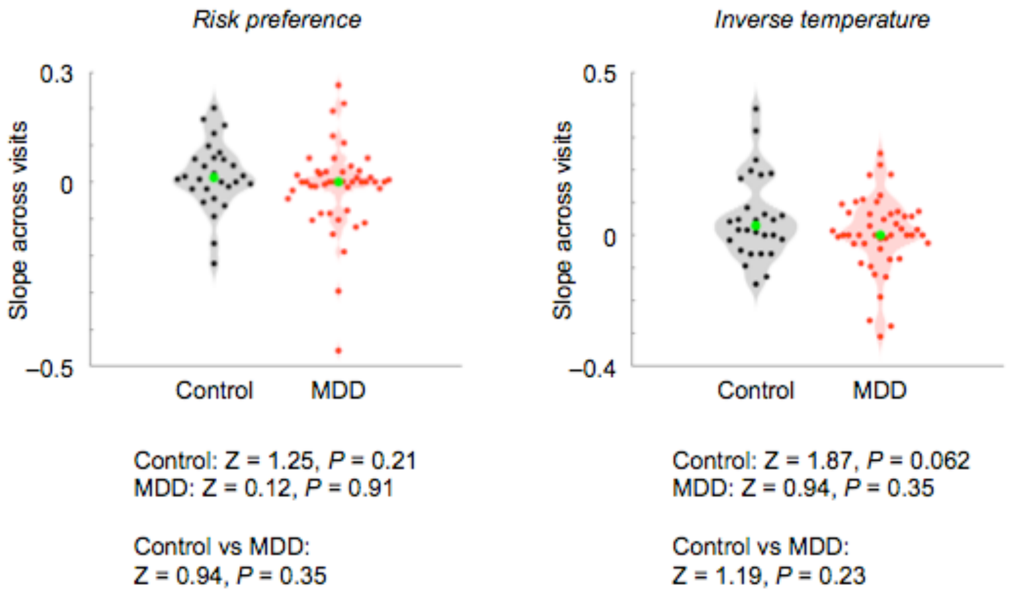

Figure. S5. Potential practice effect across visits. To directly examine whether there are changes as a function of repeated visits, the slope of changes in each individual's RP and IT across four visits were measured (linear regression). Healthy controls and individuals with MDD showed a mean slope comparable to zero (Wilcoxon's signed rank test; control: Z = 1.87, P = 0.062; MDD: Z = 0.94, P = 0.35). Although healthy controls showed a marginally positive mean slope as a group, the average slopes were not different between the groups (Wilcoxon's rank sum test, Z = 1.19, P = 0.23). These results indicate that there is no significant practice effect across repeated visits. Each point represents an individual participant, and each green dot represents the group median. Gray and red shades represent distribution of data points along the y-axis.
